# Is oligoprogression a potentially curable disease in epidermal growth factor receptor mutant lung adenocarcinoma?

**DOI:** 10.37349/etat.2023.00191

**Published:** 2023-12-13

**Authors:** Sviatoslav Chekhun, Assumpció Lopez-Paradís, Aintzane Urbizu, Teresa Morán, Anabel Mañes, Marc Cucurull, Carlos Martínez-Barenys, Iris Teruel, Gloria Moragas, Enric Carcereny, Ana Maria Muñoz Mármol, Maria Saigí

**Affiliations:** University of Campania “Luigi Vanvitelli”, Italy; ^1^Medical Oncology Department, Catalan Institute of Oncology (ICO), Badalona-Applied Research Group in Oncology (B-ARGO), Germans Trias i Pujol Research Institute (IGTP), 08918 Badalona, Spain; ^2^Pathology Department, Hospital Universitari Germans Trias i Pujol (HUGTiP), 08918 Badalona, Spain; ^3^Radiotherapy Oncology Department, Catalan Institute of Oncology (ICO)-Badalona, Hospital Universitari Germans Trias i Pujol (HUGTiP), 08918 Badalona, Spain; ^4^Thoracic Surgery Department, Hospital Universitari Germans Trias i Pujol (HUGTiP), 08918 Badalona, Spain; ^5^Diagnostic Imaging Institute (IDI), Hospital Universitari Germans Trias i Pujol (HUGTiP), 08918 Badalona, Spain

**Keywords:** Lung adenocarcinoma, epidermal growth factor receptor, oligoprogression, targeted therapy, epidermal growth factor receptor-tyrosine kinase inhibitors

## Abstract

Third-generation epidermal growth factor receptor (EGFR)-tyrosine kinase inhibitors (TKIs) have shown impressive results in *EGFR* mutant lung cancer (LC) patients in terms of disease control rate with a positive impact on overall survival. Nevertheless, after months of treatment with targeted therapy, progression inevitably occurs. Some patients develop oligoprogression and local treatment is required for optimal disease control while maintaining EGFR-TKIs. This work features a clinical case of a patient harboring an *EGFR* mutant LC undergoing oligoprogression to EGFR-TKIs, first into the brain and afterward to the primary tumor, requiring local ablative strategies, including primary tumor resection three years after the start of osimertinib. Currently, the patient is still alive and continues with a complete response upon EGFR-TKIs maintenance. Hence, oligoprogression, even in driven oncogenic tumors, represents a distinct biological entity and potential curative disease that deserves particular consideration in multidisciplinary tumor boards. In this case, tumor primary resection after three years of the initial diagnosis represents a paradigm shift in the treatment of *EGFR* mutant patients.

## Introduction

Activating oncogenic mutations in epidermal growth factor receptor (*EGFR*) are detected in up to 20% of lung adenocarcinomas (LuADCs) and are the most relevant predictor markers of response to EGFR-tyrosine kinase inhibitors (TKIs). Historically, EGFR-TKI treatment prolonged progression-free survival (PFS) in comparison to chemotherapy as an up-front treatment. Osimertinib is an irreversible third (3rd) generation EGFR-TKI that selectively inhibits both *EGFR* sensitizing mutations and the gatekeeper *EGFR T790M*, which typically arises after developing resistance to first (1st) generation and second (2nd) generation inhibitors [1]. Results from the FLAURA study showed that osimertinib significantly increases and almost doubles the median PFS in patients with previously untreated *EGFR* mutant LuADCs compared to 1st generation and 2nd generation EGFR-TKI group-hazard ratio (HR) 0.46 [95% confidence interval (CI), 0.37–0.57] [2]. In addition, osimertinib also has a greater ability to cross the blood-brain barrier and presents a higher intracranial response rate compared to the classic EGFR-TKIs (91% *vs.* 68%) [3].

However, despite a robust initial response to TKIs, acquired resistance (AR) mechanisms inevitably occur and different mechanisms have been described. This work reported a case of an *EGFR* mutant patient who underwent oligoprogression to osimertinib and benefited from local ablative strategies during the course of the disease, including resection of the primary lung tumor after three years of initial systemic treatment. The multidisciplinary approach discussed in a multidisciplinary tumor board (MTB) and the study of underlying molecular resistance mechanisms has been essential for the patient’s management.

## Case report

Here, a case report of a 53-year-old male patient, Caucasian, ex-smoker (10 packs/year), with no medical-surgical history of interest. His father died of lung cancer (LC) at the age of 67 years, and three nephews were diagnosed with breast cancer (34 years), gynecological cancer (40 years), and colorectal cancer (38 years).

**Figure 1:**
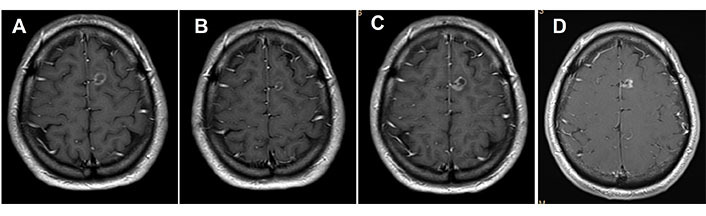
MRI of the brain. (A) Basal brain metastasis; (B) with partial response to osimertinib treatment; (C) progression after 14 cycles of osimertinib; (D) complete response after receiving SRS 20 Gy treatment

**Figure 2:**
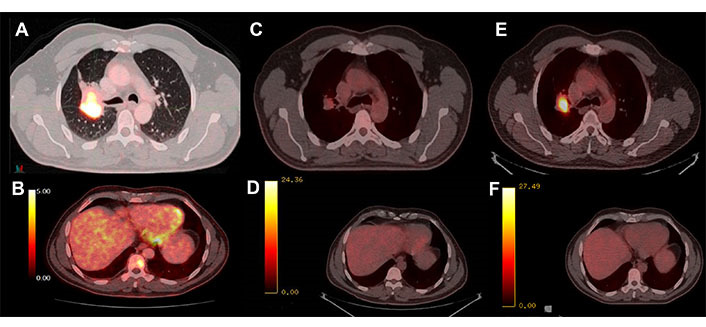
PET-CT with 18-fluorodeoxyglucose (FDG). (A) Hypermetabolism of the basal parahiliar right mass; (B) hypermetabolism of the bone metastasis at D9; (C) initial response to osimertinib in the lung mass; (D) initial response to osimertinib in the bone lesion; (E) progression of the parahiliar mass after 32 cycles of treatment; (F) complete response maintained in the bone

**Figure 3:**
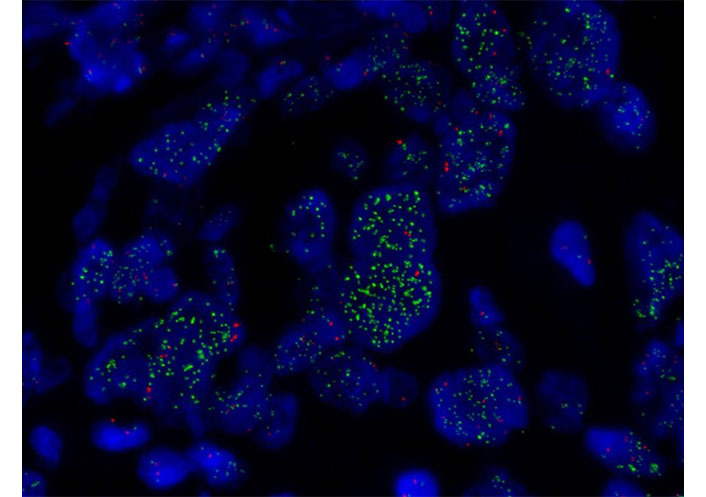
FISH of *MET*. *MET* amplification assessed by FISH (×1,000) showing *MET* high amplification in green (copy range 9–13; ratio *MET*/chromosome7: > 5)

In June 2019, he consulted for right pleuritic pain and the study was started. Chest-abdominal computed tomography (CT) revealed a 45 mm × 39 mm heterogeneous mass in the right upper lobe (RUL) and right lower paratracheal lymphadenopathy. A transbronchial biopsy was performed through bronchoscopy (BS) and pathological anatomy (PA) confirmed the diagnosis of LuADC. The molecular study by next-generation sequencing (NGS, Oncomine Solid Tumour, Life Technologies) identified 18 nucleotides deletion in exon 19 of *EGFR* (NM_005228.5): c.2240_2257del (p.Leu747_Pro753delinsSer, *EGFRdel19*). The extension study by positron emission tomography (PET)-CT and brain magnetic resonance imaging (MRI) confirmed: (1) the presence of a hypermetabolic mass in RUL with mediastinal infiltration; (2) a bone lesion in dorsal vertebra 9 (D9) confirmed by spinal MRI; and (3) a single left frontal lesion of 2 cm, compatible with brain metastasis, staging being cT2bN2M1c. In August 2019, the patient with Eastern Cooperative Oncology Group performance status (ECOG PS) 0 started treatment with osimertinib. CT scan and brain MRI for response assessment showed a reduction of the tumor in all locations, assessed as a partial response. In October 2020, cerebral oligoprogression was reported due to an increase in the frontal lesion and single-dose radiosurgery (SRS) up to 20 grays (Gy) was performed maintaining osimertinib afterward, as a systemic treatment (Figure 1). In November 2021, the CT showed an increase in the lung lesion, maintaining a complete response in the rest of the locations, including bone lesion at D9, by PET-CT (Figure 2). A BS was performed but did not obtain enough tumor material to perform a histopathological study. After discussion in the lung MTB, a right upper lobectomy and lymphadenectomy at levels 4R, 7, 9, 10, and 11R were performed in April 2022 obtaining pathological staging of LuADC ypT3N0, with negative surgical margins. Osimertinib was maintained as a systemic treatment. The NGS study by the Oncomine Comprehensive Assay panel (Life Technologies) detected the previously described *EGFRdel19,* and mesenchymal-epithelial transition (*MET*) amplification (copy range 9–13; ratio *MET*/chromosome7: > 5), which was confirmed by fluorescence *in situ* hybridization (FISH, ZytoLight^®^ SPEC MET/CEN7 Dual Color Probe, ZytoVision) (Figure 3). *MET* exon 14 skipping was also identified in a low number of reads (2,848). However, a close examination of *MET* exon 14 skipping read sequences showed two internal deletions at the end of exon 13, which are predicted to originate a non-protein productive frameshift variant. Therefore, this alteration was dismissed as a potential resistance mechanism. In addition, stop-gained mutations in tumor protein p53 (*TP53*, NM_000546.5) c.430C > T, p.Gln144Ter, variant allele frequency (VAF): 7.97%, and F-Box and WD Repeat Domain Containing 7 (*FBXW7*, NM_033632.3) c.43_44insACTC, p.Thr15AsnfsTer9, VAF: 54.16%, were also detected but not previously reported since different NGS panels were used in the two analyzed samples. Thus, whether the variants in *TP53* and *FBXW7* were detected from the beginning could not be confirmed.

Currently, the patient continues with a complete response to the primary lesion and improvement of therapeutic control of brain metastasis after almost four years from the start of osimertinib. No therapeutic modification was considered since there is no evidence of measurable active disease and the drug tolerance is excellent.

## Discussion

Osimertinib, a 3rd generation EGFR-TKI is the standard first-line treatment for metastatic patients with activating mutations in *EGFR*, according to clinical guidelines [4]. Despite the impressive results achieving long-lasting responses in a subset of patients, the emergence of AR mechanisms inevitably occurs over time.

Of note, a small proportion of LuADC patients with targetable oncogenic mutations can present oligometastatic disease at diagnosis and/or undergo oligoprogression under TKI treatment. This means that while maintaining a systemic overall response to TKI, a subset of tumor clones becomes resistant and progresses into specific locations. Once the disease is metastatic, the presence of oligoprogression suggests that addiction to the mutated oncogene still exists, although AR has occurred in some other tumor clones. However, the specific biological underlying mechanisms remain unknown [5]. In those cases, local ablative strategies such as surgery, focal radiation therapy (RT), or stereotactic body RT (SBRT), in combination with inhibitors of the altered oncogene, is a reasonable option that has shown a higher disease control rate and survival. Nevertheless, efforts to manage oligometastatic and/or oligoprogressive disease have been hampered by the lack of a uniform definition of oligoprogression, the great heterogeneity in the number and sites of disease progression, and the lack of randomized clinical trials addressing this topic [6, 7]. Therefore, a multidisciplinary approach to patient management remains essential.

In this patient with asymptomatic brain metastasis at the disease onset, it was decided to start with upfront osimertinib since high rates of intracranial response have been described and apply stereotactic radiotherapy upon focal intracranial progression [8]. Afterward, upon disease progression in the lung, surgery on the primary tumor was performed even after three years of the initial diagnosis since there was no evidence of disease in other locations. Complete response while maintaining osimertinib was maintained almost four years after the initial diagnosis. In this other recent work, the authors show a cohort of 121 oligometastatic LC patients harboring different driver mutations that have been treated with local consolidative strategies to all sites of their disease either with surgery or radiotherapy. They concluded that *EGFR* mutant patients were associated with prolonged overall survival (OS) among oligometastatic patients treated with local ablative strategies in addition to systemic therapy [9].

The molecular and histopathological reassessment of the disease, once it progresses to the EGFR-TKI contributes to a better understanding of the AR mechanisms which contributes to determining further therapeutic strategies. Mechanisms of resistance to osimertinib primarily include secondary *EGFR* mutations (e.g., C797S), *MET* and erythroblastic oncogene B-2 (*ERBB2*) amplification, or histological transformation to small cell carcinoma, among others [5].

In addition, tumor biopsies in LC, which are the most common source of cancer cells for genotyping and categorizing tumors for clinical decisions, are not always feasible. A blood-based test using cell-free circulating tumor DNA (ctDNA), also called “liquid biopsy,” represents a non-invasive, rapid, and cost-effective strategy for obtaining DNA from tumor cells. However, the technique has not yet been fully translated into clinical practice, and the variability in the amount of ctDNA released by the tumors to the bloodstream may prevent the standardization of the procedure [10]. Although no liquid biopsy was performed on our patient, dynamic approaches such as ctDNA clearance or detection of novel arising mutations in ctDNA are gaining interest in our clinical practice.

Finally, the clinical management of LC patients, as for many other cancers, would need to consider genetic predisposition as another variable. Most non-small-cell LCs (NSCLCs) with actionable oncogenic drivers, including *EGFR* mutations, are found in never-smokers and young patients. To date, the explanation is unclear, and the possibility that these cancers arise within hereditary cancer syndromes, such as the Li-Fraumeni syndrome, should be considered, particularly in *EGFR-*mutant tumors [11]. Given the family history of our patient, despite there being no major criteria for such hereditary syndrome, the study of different selected members of the family is currently ongoing but did not report any specific finding.

In summary, osimertinib remains the preferred option for patients with *EGFR* mutant advanced LC, with a significant impact on survival. However, the acquisition of resistance mechanisms is inevitable, and genetic and molecular profiling is key to advancing towards a more personalized medicine. In some cases, oligoprogression occurs and multidisciplinary management remains essential, particularly in those cases where local therapeutic strategies may prolong survival. In this case, tumor primary resection after three years of the initial diagnosis represents a paradigm shift in the treatment of *EGFR* mutant patients.

## Abbreviations

AR: acquired resistance
CT: computed tomography
ctDNA: circulating tumor DNA
D9: dorsal vertebra 9
EGFR: epidermal growth factor receptor
LC: lung cancer
LuADCs: lung adenocarcinomas
MET: mesenchymal-epithelial transition
MRI: magnetic resonance imaging
NGS: next-generation sequencing
PET: positron emission tomography
TKIs: tyrosine kinase inhibitors

## Declaration

### Availability of data and materials

The original contributions presented in the study are included in the article. Further inquiries can be directed to the corresponding author (Maria Saigí, msaigi@iconcologia.net).

### Copyright

© The Author(s) 2023.
